# TRIM26 deficiency drives gastric cancer lymph node metastasis via TGF-β signaling activation and modulates gemcitabine response

**DOI:** 10.3389/fcell.2026.1746425

**Published:** 2026-02-05

**Authors:** Kanger Shen, Daojiang Liu, Jing Su, Sirui Shen, Haiyan Zhang, Wei Xu

**Affiliations:** 1 Department of Gastroenterology, Huzhou Central Hospital, Fifth School of Clinical Medicine of Zhejiang Chinese Medical University, Huzhou, China; 2 Department of Gastroenterology, Yangxin County People’s Hospital, Huangshi, China; 3 Department of Gastroenterology, Xuzhou Central Hospital, Southeast University, Xuzhou, China; 4 Affiliated Clinical College of Xuzhou Medical University, Xuzhou, China; 5 Huzhou Central Hospital affiliated to Huzhou University, Huzhou, China

**Keywords:** chemoresistance, gastric cancer, lymph node metastasis, TGF-β signaling pathway, TRIM26

## Abstract

**Background:**

Globally, gastric cancer (GC) is a predominant cause of cancer-related death. Lymph node metastasis (LNM) and chemoresistance constitute two major barriers to improving outcomes, as LNM signifies advanced disease and chemoresistance consequently leads to treatment failure. This study systematically investigates the key molecular drivers underlying LNM and chemoresistance in GC to assess their therapeutic relevance.

**Methods:**

Our study integrated single-cell and bulk transcriptomic data from GEO and TCGA. The analytical workflow comprised: Firstly, hdWGCNA for co-expression network construction; Secondly, a combination of machine learning algorithms (LASSO, random forest, and SVM-RFE) for core gene screening; Thirdly, pseudotime trajectory analysis (Monocle2/3) to delineate cell state transitions. Cell-cell communication and metabolic pathways were profiled using CellChat and scMetabolism, respectively. Computational pharmacology involved drug sensitivity prediction with the pRRophetic algorithm, complemented by molecular docking and dynamics simulations for structural insights. Finally, TRIM26’s functional roles were experimentally validated through CCK-8, Transwell, and colony formation assays, alongside protein-level verification by immunohistochemistry.

**Results:**

Downregulation of TRIM26 in GC correlated strongly with LNM and poor survival. At single-cell resolution, TRIM26 loss in epithelial cells fueled pro-metastatic crosstalk with endothelial cells and macrophages through SELE-CD44 and SPP1-CD44/integrin axes. This triggered TGF-β activation, TP53 network dysregulation, and metabolic reprogramming of taurine and pantothenate/CoA pathways. TRIM26-low tumors were predicted to be less sensitive to gemcitabine, consistent with higher estimated IC_50_ values, a premise bolstered by computational evidence of stable, direct drug binding (free energies: −6.7 and −5.5 kcal/mol) and sustained interactions in 100 ns simulations. Critically, TRIM26 overexpression curtailed tumor growth and invasiveness in the presence of gemcitabine.

**Conclusion:**

TRIM26 inhibits LNM by modulating TGF-β signaling and remodeling the tumor microenvironment. Clinically, low TRIM26 expression identifies tumors with reduced sensitivity to gemcitabine, reflected by higher estimated IC_50_ values—a correlation underpinned by computational models demonstrating stable drug binding. Thus, TRIM26 serves as a integrated prognostic and predictive biomarker, positioning it as a promising theranostic target to inform precision therapy strategies in GC.

## 1 Introduction

GC is a leading digestive tract malignancy worldwide. According to Global Cancer Statistics 2022 (Bray et al., 2024), it ranks fifth in incidence and fourth in mortality, posing a significant global health burden. The disease exhibits a distinct geographical predominance, with incidence and mortality rates in East Asia (including China, Japan, and South Korea) substantially higher than in Western nations. While advancements in endoscopic screening, surgical techniques, and targeted therapies have improved outcomes for early-stage patients, most cases are diagnosed at advanced stages with limited treatment options (Yu and Mehta, 2025). Consequently, the 5-year survival rate for advanced GC remains below 30%, notably lower than that of other gastrointestinal malignancies (Yu and Mehta, 2025). This stark prognosis underscores the urgent need to elucidate the mechanisms of GC metastasis, particularly LNM, which is critical for improving patient outcomes (Chen et al., 2024; Deng et al., 2025).

LNM is the predominant and initial pathway of dissemination in GC, critically influencing TNM staging, surgical planning, and adjuvant therapy (Zhu et al., 2023). Clinical evidence consistently links the burden of metastatic lymph nodes to overall survival (OS), with LNM-positive patients experiencing significantly higher recurrence and worse long-term outcomes. The prognostic disparity is stark: the 5-year survival rate drops below 20% in extensively node-positive cases, compared to over 60% in node-negative patients (Lianos et al., 2019). Understanding the molecular basis of LNM is therefore essential for improving risk stratification and guiding precision therapy (Lianos et al., 2019). Mechanistically, GC lymphatic spread is a multistep process driven by dynamic crosstalk between tumor cells and the tumor microenvironment (TME) (Chen et al., 2014). Key events include epithelial-mesenchymal transition (EMT), which enhances cell motility and invasiveness; extracellular matrix remodeling via protease secretion; and angiogenesis/lymphangiogenesis that opens routes for dissemination (Shi et al., 2019). Immune evasion further supports metastasis, as tumor-associated macrophages, regulatory T cells, and other immunosuppressive components secrete cytokines to dampen antitumor immunity (Wang et al., 2023a). Additionally, metabolic reprogramming—such as dysregulation of glutamine and lipid metabolism—supplies energy and biosynthetic precursors to support metastatic progression (Song et al., 2017). Thus, GC LNM arises not from isolated genetic alterations, but from the convergence of multiple signaling pathways and cellular interactions. While pathways like TGF-β, PI3K/AKT, and Wnt/β-catenin are well-established contributors, the core regulatory network governing LNM remains incompletely elucidated, and clinically applicable molecular drivers are still lacking (Chen et al., 2022).

The tripartite motif (TRIM) family of E3 ubiquitin ligases plays a central role in ubiquitination, regulating diverse processes including protein degradation, DNA damage repair, immune signaling, and cell-cycle progression (Wu et al., 2024; Jiang et al., 2024). Comprising characteristic RING, B-box, and coiled-coil domains, TRIM proteins exhibit dual functionalities in cancer pathogenesis. For instance, TRIM28 and TRIM59 often act as oncoproteins by enhancing proliferation and metastasis, whereas TRIM16 and TRIM62 function as tumor suppressors by inhibiting growth and promoting apoptosis (Ma et al., 2023; Han et al., 2018; Li et al., 2023a; Fan et al., 2022). Among these, TRIM26 represents a context-dependent regulator with tissue-specific roles. It displays oncogenic activity in hepatocellular carcinoma by facilitating p53 degradation and impeding apoptosis, while in antiviral immunity, it suppresses the STING-IRF3 pathway (Li et al., 2024)^.^ Proteomic and transcriptomic analyses further associate aberrant TRIM26 expression with tumor invasion and immune evasion in various cancers (Li et al., 2021). Despite these insights, the function of TRIM26 in GC—particularly its influence on LNM—remains largely unexplored, highlighting its potential as a key target for elucidating novel metastatic mechanisms in GC.

Single-cell RNA sequencing (scRNA-seq) has transformed tumor biology by enabling high-resolution analysis of cellular heterogeneity, functional states, and developmental trajectories—capabilities beyond the reach of bulk transcriptomic profiling (Li et al., 2022). In GC, scRNA-seq has unveiled tumor evolution and microenvironmental dynamics, such as shifts in immunosuppressive cell composition, activation of cancer-associated fibroblasts, and remodeling of the pre-metastatic niche in lymph nodes (Su et al., 2024). When integrated with analytical methods like WGCNA, machine learning (LASSO, random forest, SVM-RFE), pseudotime inference, and cell-cell communication analysis, scRNA-seq provides a powerful framework for systematically identifying key genes and signaling networks that drive metastatic progression.

This study aims to elucidate the dual role of TRIM26 in driving LNM and modulating drug response. Through integrated analysis of single-cell and bulk transcriptomic data, we will delineate the TRIM26-centered regulatory network, characterize its functional impact on metastasis and tumor microenvironment remodeling, and pinpoint TRIM26-associated chemotherapeutic agents. These insights will critically evaluate TRIM26’s potential as a clinical biomarker and therapeutic target.

## 2 Materials and methods

### 2.1 Data acquisition and preprocessing

The scRNA-seq dataset was sourced from the GEO database (accession: GSE163558), comprising three primary GC tissues, one adjacent normal tissue, and six metastatic GC samples—including two LNM. From these, three primary GC and two LNM tissues were selected for downstream analysis to ensure comparability.

Data preprocessing was conducted using the Seurat R package. Initial quality control involved filtering cells with expression of fewer than three genes and genes detected in fewer than 50 cells. These steps effectively reduced technical noise and background signal, resulting in a high-quality expression matrix for subsequent analytical steps.

Bulk RNA-seq training data were obtained from the TCGA-STAD project, restricted to stomach-derived adenomas and adenocarcinomas with available transcriptome profiling (Gene Expression Quantification). Clinical variables such as age, sex, and race were not used as exclusion criteria.Gene-level expression values were consolidated by averaging multi-probe measurements, and samples with no detectable expression were removed. The data were uniformly processed using the Toil pipeline, converted to FPKM and then TPM format, and finally log2-transformed to improve comparability. Corresponding clinical data were curated, and patients under 18 years of age or with survival times less than 30 days were excluded to enhance clinical relevance.

### 2.2 Processing and visualization of scRNA-seq data

scRNA-seq data were processed using Seurat (v4.0.0). A two-step filtering strategy was applied to remove low-quality cells and potential doublets. Genes detected in fewer than 3 cells and cells expressing fewer than 250 genes were excluded. Further quality control was performed based on the following thresholds:(1) Retain cells with 500–6000 detected genes; (2) Include cells with UMIs >1,000, excluding the top 3% by UMI count; Exclude cells with mitochondrial gene content >35% and remove the top 2% with highest mitochondrial expression; (3) Remove cells in the top and bottom 1% by ribosomal RNA proportion; (4) Retain cells with total RNA counts >1,000, excluding the top 3% by total RNA. To further ensure data quality by removing potential doublets (multiple cells captured within a single droplet), we performed doublet detection using the DoubletFinder R package (v2.0.3) with an estimated doublet formation rate of 10%. Predicted doublets were removed from subsequent analyses. After filtering, expression data were normalized using the NormalizeData function, and highly variable genes were selected via FindVariableFeatures. Principal component analysis (PCA) was performed for dimensionality reduction, and batch effects were corrected using the Harmony algorithm with “orig.ident” as the covariate. Convergence was assessed using diagnostic plots. UMAP was applied for visualization based on the top 30 principal components. Cell clustering was conducted using FindNeighbors and FindClusters, with cluster identities assigned via Idents.

### 2.3 Single-cell RNA sequencing analysis and cell type annotation

Single-cell RNA sequencing data were processed using a standard analysis workflow. Low-quality cells were filtered out based on the number of detected genes and mitochondrial gene expression. After normalization and identification of highly variable genes, dimensionality reduction was performed by principal component analysis (PCA), followed by unsupervised clustering using a graph-based clustering algorithm.

Cell subpopulations were annotated according to the expression of well-established canonical marker genes reported in previous gastric cancer single-cell studies. Specifically, epithelial cells were identified by high expression of EPCAM and KRT19; malignant epithelial cells were further characterized by elevated EPCAM, KRT8, and KRT18 expression. T cells were defined by CD3D and CD3E expression, with CD4^+^ and CD8^+^ T cell subsets identified by CD4 and CD8A, respectively. B cells were annotated based on MS4A1 and CD79A expression, while plasma cells were identified by high levels of MZB1 and XBP1. Myeloid cells were characterized by LST1 and TYROBP expression, with macrophages defined by CD68 and C1QC, and dendritic cells by FCER1A and CLEC10A. Fibroblasts were identified using COL1A1 and COL1A2, and endothelial cells by PECAM1 and VWF.

CopyKAT analysis revealed that EPCAM-positive epithelial clusters exhibited extensive aneuploidy, confirming their malignant nature. These malignant epithelial cells were therefore defined as gastric cancer cells and used for downstream analyses.

### 2.4 Identification of LNM-associated genes in GC cells using hdWGCNA

To identify key gene modules associated with LNM in GC, we performed high-dimensional weighted gene co-expression network analysis (hdWGCNA). A high-quality expression matrix was prepared using SetupForWGCNA, and metacells were generated via MetacellsByGroups (k = 25, max_shared = 10) to improve data robustness. The metacell matrix was normalized, standardized, and integrated using PCA and Harmony, followed by UMAP visualization. A co-expression network was constructed after determining the optimal soft-thresholding power with TestSoftPowers. Module eigengenes were calculated using ModuleEigengenes to summarize expression patterns. Differential expression analysis between primary and LNM samples was conducted with FindMarkers (P < 0.05). Candidate LNM-associated genes were defined as the intersection of significant differentially expressed genes (DEGs) and hub genes identified from relevant WGCNA modules.

### 2.5 Machine learning-based screening of LNM-related genes

To identify robust gene signatures associated with LNM, we employed a multi-step feature selection approach. First, univariate Cox regression was used to screen for metastasis-associated genes with prognostic significance. Subsequently, three distinct machine learning algorithms were applied to refine the gene set: (1) LASSO regression via the glmnet package to perform feature selection with regularization; (2) SVM-RFE (e1071 package) for recursive feature elimination based on support vector machine weights; (3) random forest (randomForest package) to rank genes by mean decrease Gini importance. The final candidate genes were defined as the intersection of features selected by all three methods, followed by validation through survival analysis and expression pattern assessment.

### 2.6 Evaluation of expression patterns, diagnostic, and prognostic value

The expression profiles of candidate genes were systematically evaluated across normal gastric mucosa, primary GC, and LNM tissues from the TCGA cohort. To assess the prognostic relevance of candidate genes, univariate and multivariate Cox proportional hazards regression analyses were performed. Relevant clinicopathological variables, including age, gender, tumor stage, and histological grade, were incorporated into the multivariate model to adjust for potential confounding factors, thereby determining whether candidate genes served as independent prognostic indicators.

### 2.7 Pathway analysis of downstream signaling

Downstream signaling pathways were investigated using two complementary computational approaches: TCGA-STAD samples were stratified by median expression of key genes into high- and low-expression groups. DEGs were identified using limma (∣log_2_FC∣ >1, FDR <0.05). Concurrently, WGCNA was performed on the top 25% most variable genes to construct co-expression networks, with soft thresholding power optimized for scale-free topology. Gene modules significantly correlated with LNM (P < 0.05, ∣correlation coefficient∣ >0.3) were selected, and their overlap with DEGs was used for KEGG pathway enrichment analysis via clusterProfiler (P < 0.05). Mfuzz soft clustering was applied to the top 5,000 high-variance genes from TCGA samples (c = 50 clusters). Expression dynamics were tested for association with LNM status using χ^2^ tests or logistic regression. Core genes from LNM-associated clusters underwent KEGG enrichment analysis to identify phenotype-relevant pathways.

### 2.8 Pseudotime trajectory analysis

Cell state transitions during GC LNM were reconstructed using pseudotime analysis in Monocle2 and Monocle3. In Monocle2, a CellDataSet was created followed by estimation of size factors and dispersions. Genes with mean expression ≥0.1 were retained, and the top 200 highly variable genes were selected for DDRTree dimensionality reduction. Cells were ordered along the trajectory using orderCells, and BEAM analysis identified branch-dependent differentially expressed genes (P < 0.05), with particular focus on transcription factors and signaling components. For validation, Monocle3 was employed with UMAP-based reduction, learn_graph for trajectory inference, and graph_test to assess spatial autocorrelation (Moran’s I). This dual-framework approach ensured robust identification of transcriptional programs driving metastatic progression.

### 2.9 Analysis of cell-cell communication

CellChat was employed to characterize intercellular signaling networks within the tumor microenvironment. Using its curated ligand-receptor database integrated with protein-protein interaction networks, we computed communication probabilities and quantified pathway-level information flow. Cell populations were classified into distinct functional roles (sender, receiver, mediator, and influencer) based on their signaling patterns. Comparative analysis between primary and LNM tissues identified metastasis-associated alterations in cell-cell communication.

### 2.10 Inference of transcription factor activity

Transcription factor (TF) activities were computationally inferred by integrating the DoRothEA database with the Viper algorithm. High-confidence regulatory interactions (confidence levels A–C) were used to construct regulons, whose activity scores were calculated per cell and visualized through PCA and UMAP. Differential TF activity analysis across cell populations was performed and visualized using hierarchical clustering (pheatmap). Protein-protein interaction networks were built from significantly altered TFs, and hub regulators were identified via MCODE in Cytoscape.

### 2.11 Analysis of metabolic reprogramming

Metabolic pathway activities were quantified at single-cell resolution using scMetabolism, which applies ssGSEA and AUCell algorithms to score key processes including glycolysis, fatty acid metabolism, amino acid metabolism, and the TCA cycle. Comparative analyses were performed between primary and LNM samples, as well as between high- and low-expression groups of candidate genes. Pathways consistently altered across these comparisons were identified as core metabolic features associated with metastatic progression.

### 2.12 Molecular docking

Molecular docking was performed to evaluate potential binding between TRIM26 and the chemotherapeutic agents gemcitabine and paclitaxel. The three-dimensional structures of the small molecules were obtained from PubChem and optimized using ChemOffice. The crystal structure of TRIM26 was retrieved from the RCSB PDB database and prepared by removing non-protein components with PyMOL 2.6. Using AutoDock 1.5.6, hydrogen atoms were added and rotatable bonds were assigned to the ligands. Docking simulations were conducted with AutoDock Vina, with binding site-specific coordinates defining the search space. The conformation with the most favorable binding energy was selected, where values below −5.0 kcal/mol and −7.0 kcal/mol indicate substantial and high-affinity binding, respectively. Protein–ligand interactions were visualized in Discovery Studio 2019 and PyMOL 2.6.

### 2.13 Molecular dynamics simulation

Molecular dynamics (MD) simulations were conducted with GROMACS 2022 to assess the dynamic stability of TRIM26 in complex with gemcitabine and paclitaxel. The TRIM26 structure was modeled using the amber14sb force field, while ligand parameters were derived from GAFF2 via the AutoFF server. Each protein–ligand complex was solvated in a TIP3P water box (1.0 nm boundary) and neutralized with ions. Electrostatic interactions were computed using the Particle Mesh Ewald method with a 1.0 nm cutoff. Bonds were constrained via the LINCS algorithm, and a 1 fs time step was applied using the Verlet leap-frog integrator.

The system underwent energy minimization (3,000 steps steepest descent, 2000 steps conjugate gradient) prior to a 100 ns production simulation under NPT conditions (310 K). Trajectories were analyzed for root mean square deviation (RMSD), root mean square fluctuation (RMSF), radius of gyration (Rg), solvent accessible surface area (SASA), and hydrogen bond count to evaluate conformational stability and binding interactions.

### 2.14 Cell culture

The human GC cell lines AGS and HGC-27, sourced from the Cell Resource Center of Beijing Union Medical College, were cultured in DMEM or RPMI-1640 medium (HyClone, United States, #SH30809.01, SH30022.01) containing 10% fetal bovine serum (Gibco, United States, #10099-141) and 1% penicillin–streptomycin (Gibco, United States, #15140122). Cells were maintained at 37 °C under 5% CO_2_ in a humidified atmosphere and passaged at a 1:3 ratio every 3 days.

### 2.15 CCK-8 assay

Cell viability was measured using the Cell Counting Kit-8 (Dojindo, Japan, #CK04). Cells were seeded in 96-well plates at 2–5 × 10^3^ cells per well (3–5 replicates per group) and allowed to adhere for 12–24 h. After treatment with gemcitabine (MCE, United States, # HY-17026) at concentrations of 0.2, 0.4, 0.6, and 0.8 μmol/L, 10 μL of CCK-8 reagent was added to each well and incubated for 4 h. Absorbance at 450 nm was recorded using a microplate reader (BioTek Synergy H1). Lastly, the IC_50_ value was calculated by GraphPad Prism software, employing a four-parameter logistic regression model. The model equation is y = A2 + (A1 - A2)/(1 + (x/IC_50_) ^ p), where y denotes cell viability, x is the inhibitor concentration, A1 and A2 correspond to the maximum and minimum viability values, respectively, and p stands for the slope factor.

### 2.16 Lentiviral transfection

Lentivirus for TRIM26 overexpression was purchased from GenePharma Co., Ltd. (Shanghai, China). When the confluency of AGS and HGC27 cells reached around 20%, the lentivirus was transduced into the cells at a multiplicity of infection (MOI) of 1:30 with polybrene added to enhance infection efficiency. The culture medium was refreshed 24 h post-transduction, and 48 h after transduction, puromycin-containing medium was added for selection. Cells that survived puromycin selection were considered stably transduced clones, and the transduction efficiency was verified by Western blot analysis.

### 2.17 Colony formation assay

Cells were plated in 6-well plates at 500–1,000 cells per well (triplicate wells per group) and cultured for 10–14 days. Resulting colonies were fixed with 4% paraformaldehyde (Beyotime, China #P0099), stained with crystal violet (Beyotime, #C0121), and quantified after imaging.

### 2.18 Transwell assay

Cell migration capacity was evaluated via Transwell chamber assays (Corning, United States, # 353097). Briefly, a total of 3 × 10^4^ cells suspended in 400 μL serum-free medium were seeded into the upper compartment of the chamber. For the invasion assay, Matrigel was diluted at a ratio of 1:15 and pre-coated onto the upper chamber prior to cell seeding. Meanwhile, the lower chamber was filled with complete medium containing 20% FBS to serve as a chemoattractant. Following 24 h of incubation, cells that had not migrated were carefully wiped off from the upper surface of the membrane. Migrated cells adhering to the lower side of the membrane were fixed with 4% paraformaldehyde, stained with crystal violet, and finally observed and photographed under an inverted microscope (Nikon, Tokyo, Japan).

### 2.19 Western blot

Cells were lysed using RIPA lysis buffer containing phosphatase and protease inhibitors (Beyotime, #P0013, P1045). The supernatant was collected after centrifugation and the protein concentration was determined by the BCA kit (Beyotime, #P0011). Then, the protein samples were mixed with loading buffer and denatured at 95 °C for 5 min before loading. Protein (30 μg) separation was achieved by SDS-PAGE electrophoresis (NCM Biotech, China, #P2012), followed by transfer to a membrane (GE Healthcare Life Science, Germany, #10600023). After blocking non-specific binding sites, the membrane was incubated with the primary antibody overnight at 4 °C. The next day, after washing, the membrane was incubated with the corresponding secondary antibody, and finally chemiluminescence was used for imaging (NCM Biotech, #10,100). The following are the antibodies used for Western blot analysis: TRIM26 (Proteintech, United States, #27013-1-AP), GAPDH (HUABIO, China, #ET1601-4), TGF-β1 (HUABIO, #HA721143), P-Smad2/3 (abcam, #ab254407), Smad2/3 (abcam, #ab207447).

### 2.20 Statistical analysis

Statistical analyses of all data were performed using GraphPad Prism 9.0. For data following a normal distribution, Student’s t-test was applied; for non-normally distributed data, the Wilcoxon rank-sum test was used instead. *P < 0.05, **P < 0.01, and ***P < 0.001.

## 3 Results

### 3.1 Single-cell transcriptomic landscape of the GC immune microenvironment

**FIGURE 1:**
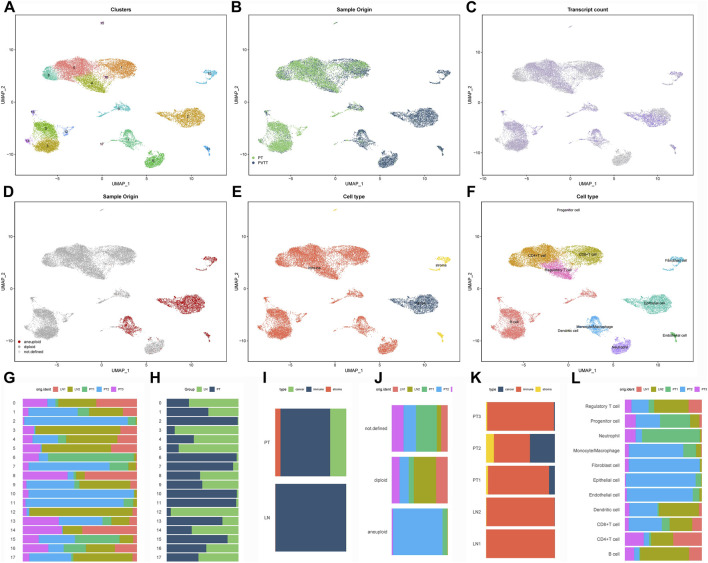
Single-cell transcriptomic landscape of the GC immune microenvironment **(A)** UMAP visualization of 18 transcriptionally distinct cell clusters identified through integrated analysis of primary gastric tumors and matched lymph node metastases. **(B)** Tissue origin mapping of single cells, distinguishing between primary tumor (red) and lymph node metastatic (blue) populations. **(C)** Cellular transcriptional activity reflected by the number of detected genes per cell (nFeature), displayed across clusters. **(D)** Copy number variation landscape inferred by CopyKAT, revealing aneuploid tumor cells that largely coincide with EPCAM-positive epithelial populations. **(E)** Preliminary cell type classification based on established lineage markers. **(F)** Refined annotation identifying eight major cellular compartments through integrated computational (SingleR, CellMarker) and manual curation approaches. **(G–L)** Cellular composition analysis: cluster-wise **(G,H)** and cell type-specific **(I,J)** proportional comparisons between primary and metastatic sites, with sample-level distribution shown in panels **(K,L)**.

Following stringent quality control, 22,450 high-quality cells expressing 24,577 genes were retained from primary GC lesions and matched lymph node metastases. Data normalization, principal component analysis (top 30 PCs), and UMAP-based nonlinear dimensionality reduction identified 18 transcriptionally distinct cell clusters (Figure 1A). The dataset comprised 11,442 cells from primary tumors and 11,008 from metastatic lymph nodes, revealing compositional differences between sites (Figure 1B). Transcriptional heterogeneity was evident across clusters, with clusters 0, 2, 4, and seven displaying high global gene expression, whereas clusters 6, 8, and 14 showed comparatively lower activity (Figure 1C).

CopyKAT-based inference of copy number alterations (CNAs) identified aneuploid populations that largely overlapped with EPCAM-positive clusters, confirming their malignant epithelial origin (Figure 1D). Using canonical marker genes combined with multi-method annotation (SingleR, CellMarker database, and manual curation), we classified the 18 subclusters into eight major lineages, including epithelial, immune, fibroblast, and endothelial cells (Figures 1E,F). Comparative analysis revealed shifts in the relative abundance of these cell types between primary and metastatic sites (Figures 1G–L), underscoring dynamic remodeling of the tumor microenvironment during lymphatic spread.

### 3.2 Identification of gene modules associated with LNM using hdWGCNA

**FIGURE 2:**
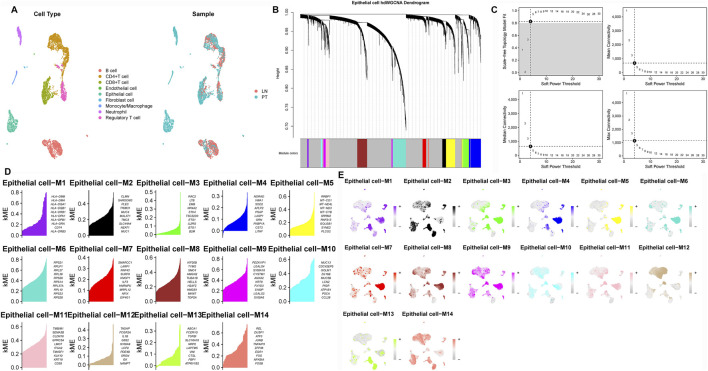
Construction of the hdWGCNA network in gastric cancer **(A)** Analysis of network topology for various soft-thresholding powers. **(B)** Scale-free topology fit (left y-axis) and mean connectivity (right y-axis) as functions of soft-thresholding power, demonstrating the selection of power four for subsequent analysis. **(C)** Validation of the chosen soft-thresholding power (β = 4) achieving approximate scale-free topology. **(D)** Gene dendrogram and module assignment based on topological overlap matrix (TOM), with colors representing distinct co-expression modules (gray module excluded). **(E)** Heatmap illustrating module eigengene enrichment patterns across identified cell clusters, revealing cell type-specific co-expression characteristics.

**FIGURE 3:**
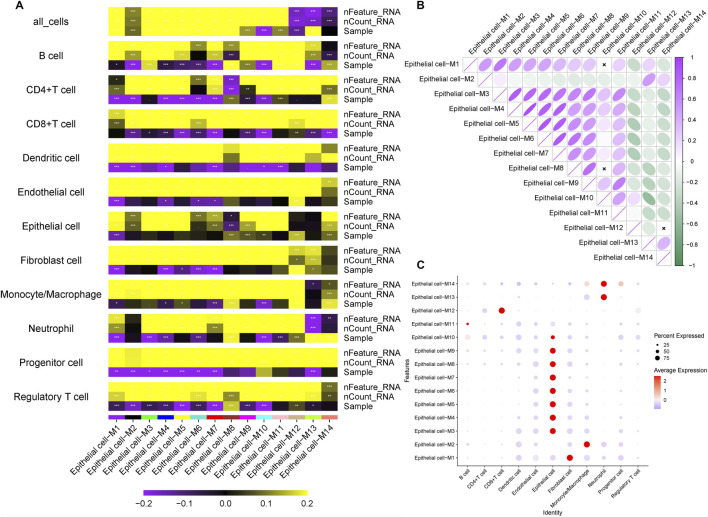
Correlation between WGCNA modules and cell types **(A)** Module-cell type correlation matrix identifies M3 and M5 as epithelial-specific modules, showing significant positive correlation with epithelial cell signatures. **(B)** Intermodular correlation patterns reveal co-expression relationships among distinct gene modules. **(C)** Eigengene-based correlation profiling confirms preferential association of specific modules with defined cellular compartments.

To identify epithelial gene modules linked to LNM, we performed weighted gene co-expression network analysis (hdWGCNA) on the single-cell expression matrix using a soft-threshold power of 4. This revealed 14 distinct co-expression modules (Figures 2A–D). Among these, the M3 and M5 modules exhibited strong correlations with epithelial cell identity (Figure 3A) and collectively contained 127 hub genes significantly enriched in epithelial subclusters (Figure 2E). Despite some inter-module co-expression, M3 and M5 maintained relatively independent expression patterns (Figure 3B), and their strong epithelial association was further corroborated by correlation analysis (Figure 3C). These results indicate that M3 and M5 represent key functional units potentially governing epithelial behavior and metastatic propensity in GC.

### 3.3 Screening of candidate genes for LNM and prognostic relevance

**FIGURE 4:**
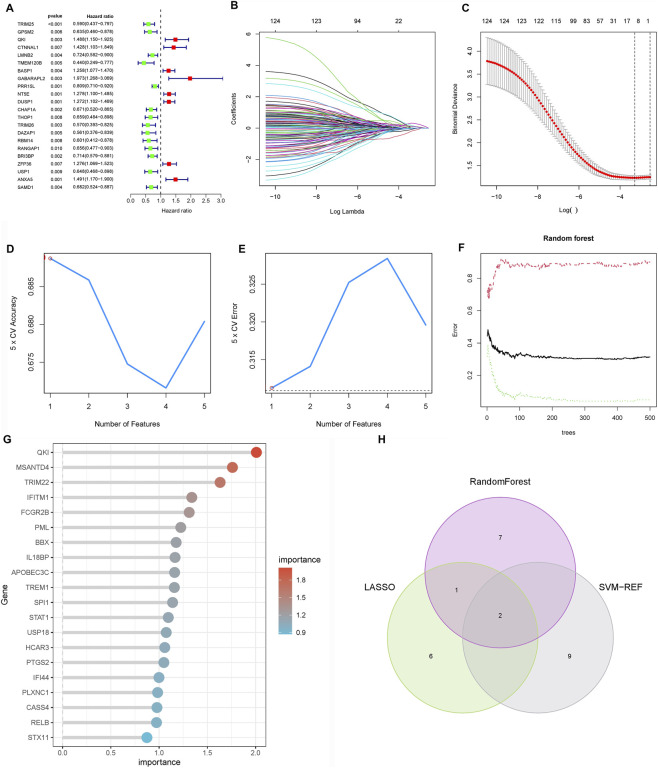
Screening of candidate genes for LNM and prognostic relevance **(A)** Univariate Cox regression analysis of 127 candidate genes reveals 22 with significant association with overall survival. **(B)** LASSO regression coefficient profiles across lambda values. **(C)** Cross-validation curve identifying the optimal penalization parameter for LASSO regression, resulting in nine prognostic genes. **(D)** Random forest variable importance ranking by mean decrease accuracy. **(E)** Model error rates relative to tree number in random forest analysis, highlighting ten top-ranked genes. **(F)** SVM-RFE average predictive accuracy across feature subsets. **(G)** Identification of the optimal feature number in SVM-RFE evaluation, yielding 11 candidate genes. **(H)** Integrative screening identifies TRIM26 and IRAK2 as consensus prognostic markers shared by all three machine learning approaches.

To identify prognostic drivers of LNM, we first performed univariate Cox regression on the 127 hub genes from the M3 and M5 modules, identifying 22 genes significantly associated with overall survival (P < 0.05; Figure 4A). To enhance robustness, we applied three machine learning algorithms: LASSO regression selected nine prognostic genes (Figures 4B,C), random forest ranked the top 10 features (Figures 4D,E), and SVM-RFE identified 11 predictors (Figures 4F,G). Cross-algorithm comparison revealed TRIM26 and IRAK2 as consistently selected candidates (Figure 4H). Further validation in the TCGA-STAD and GEO cohorts showed inconsistent prognostic relevance for IRAK2, leading to its exclusion. In contrast, TRIM26 consistently correlated with LNM and poor survival. To eliminate the potential impact of confounding factors on the prognostic value of TRIM26, a multivariate Cox regression analysis was performed. The results demonstrated that, after adjusting for age, sex, tumor stage, and grade, TRIM26 remained significantly associated with patient prognosis (P = 0.005), indicating that TRIM26 is an independent prognostic factor (Supplementary Figure S2).

### 3.4 TGF-β signaling is a key downstream pathway regulated by TRIM26

**FIGURE 5:**
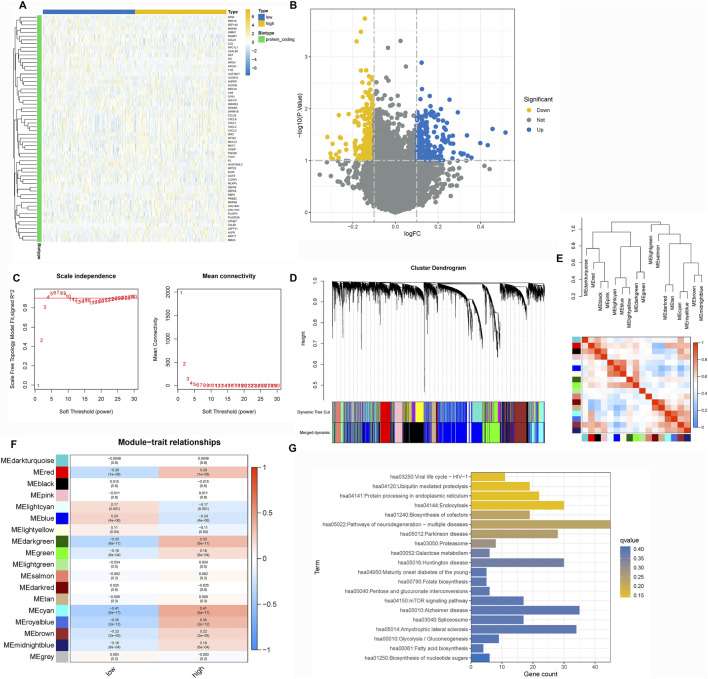
TGF-β signaling is a key downstream pathway regulated by TRIM26 **(A)** Volcano plot displaying differentially expressed genes between TRIM26-high and TRIM26-low GC samples. **(B)** Hierarchically clustered heatmap of DEGs showing distinct transcriptional profiles between TRIM26 expression groups. **(C)** Gene clustering dendrogram illustrating co-expression module assignments. **(D)** Eigengene dendrogram demonstrating relationships among identified modules. **(E)** Module-trait correlation heatmap highlighting the cyan module as most strongly associated with TRIM26 expression levels. **(F)** Gene interaction network of the cyan module, representing key TRIM26-correlated genes. **(G)** KEGG pathway enrichment analysis of genes overlapping between DEGs and the cyan module.

**FIGURE 6:**
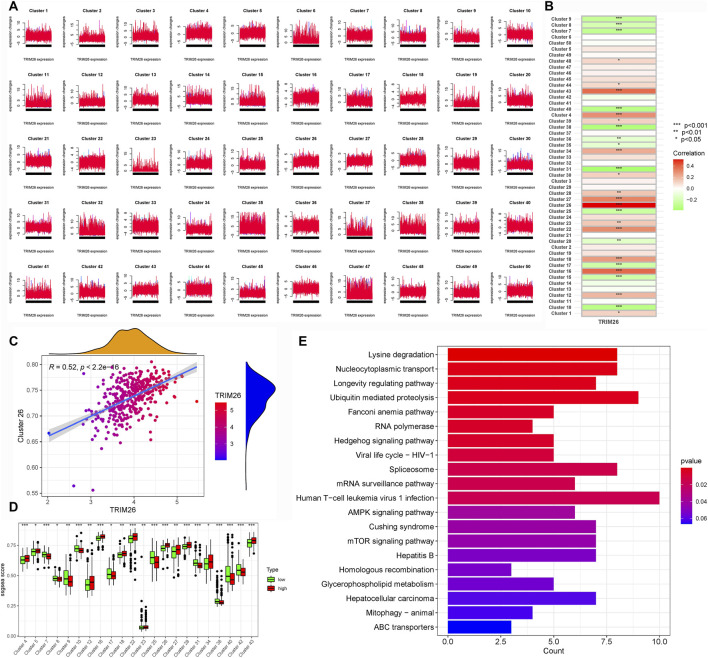
TRIM26 co-expression patterns identified by Mfuzz soft clustering **(A)** Mfuzz-based classification of genome-wide expression dynamics across GC samples, organized into 50 distinct temporal patterns. **(B)** Expression trajectory profile of Cluster 26, showing the most significant positive correlation with TRIM26 levels. **(C)** Centroid expression pattern characterizing the core transcriptional behavior of Cluster 26. **(D)** Representative gene members comprising the TRIM26-associated Cluster 26 program. **(E)** Functional annotation of Cluster 26 genes through KEGG pathway enrichment analysis.

To delineate the molecular mechanisms by which TRIM26 influences LNM, we integrated WGCNA and Mfuzz soft clustering analyses. Differential expression analysis between TRIM26-high and TRIM26-low cells identified 513 significantly dysregulated genes (∣log_2_FC∣ >1, adjusted P < 0.05; Figures 5A,B). WGCNA further revealed a cyan module comprising 861 genes that showed the strongest positive correlation with TRIM26 expression (R > 0.6, P < 0.01; Figures 5C–F). Integration of these genes with the DEGs yielded 1,374 candidate targets, which were enriched in mTOR signaling, TGF-β signaling, and cell adhesion molecules (Figure 5G). In parallel, Mfuzz soft clustering identified 50 expression pattern clusters, among which cluster 26 (442 genes) was most strongly correlated with TRIM26 expression (Pearson R > 0.5, P < 0.01; Figures 6A–D). KEGG enrichment of this cluster again highlighted TGF-β, Hedgehog, and Wnt signaling pathways (Figure 6E). Collectively, these complementary analyses consistently identify TGF-β signaling as a major downstream pathway through which TRIM26 regulates GC metastasis.

### 3.5 Pseudotime trajectory analysis links TRIM26 downregulation to early tumor differentiation states

**FIGURE 7:**
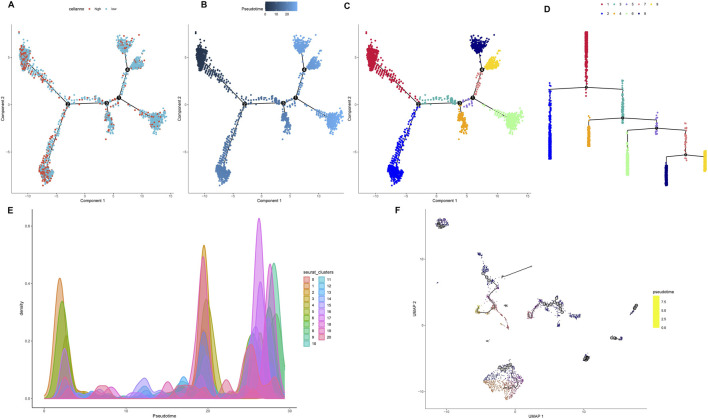
Pseudotime trajectory analysis links TRIM26 downregulation to early tumor differentiation states **(A)** Developmental trajectory reconstructed by Monocle2, depicting cellular branching structure and lineage paths. **(B)** Pseudotime mapping revealing nine distinct differentiation trajectories in GC cells. **(C)** Spatial distribution of proliferative cells (expressing PCNA and MKI67) localized at the trajectory origin. **(D)** TRIM26 expression gradient along developmental pseudotime, showing enrichment in early, less-differentiated branches. **(E)** Monocle3-based trajectory analysis confirming TRIM26 expression patterning. **(F)** Spatial autocorrelation analysis (Moran’s **(I)** validating the significance of TRIM26 distribution along developmental paths.

Developmental trajectory reconstruction using Monocle2 revealed GC cells organized along four branching paths, delineating nine distinct differentiation routes that reflect substantial lineage heterogeneity (Figures 7A,B). Cells exhibiting high proliferation marker expression (PCNA, MKI67) localized to the trajectory origin, corresponding to poorly differentiated states (Figure 7C). Spatial mapping of TRIM26 expression showed enrichment of TRIM26-low populations in early branching regions, particularly branch 1, while TRIM26-high cells clustered in later trajectory segments (Figure 7D). This spatial distribution suggests an association between reduced TRIM26 expression and less differentiated, more aggressive phenotypic states. Branch Expression Analysis Modeling (BEAM) identified TRIM26 as dynamically regulated across branching points (P < 0.05). These findings were corroborated by Monocle3, which similarly demonstrated elevated TRIM26 expression in early pseudotime followed by progressive downregulation. Spatial autocorrelation analysis (Moran’s I = 0.02, P < 0.05; Figures 7E,F) further supported a statistically significant patterning of TRIM26 along developmental trajectories.

### 3.6 TRIM26-deficient epithelial cells drive microenvironmental crosstalk in metastatic niches

**FIGURE 8:**
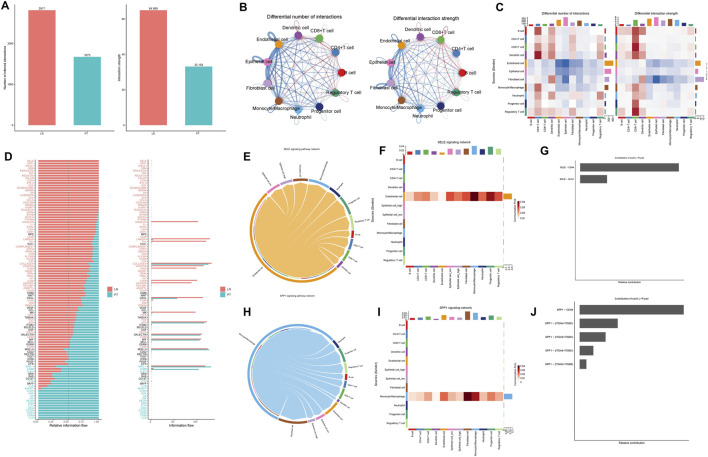
TRIM26 deficient epithelial cells drive microenvironmental crosstalk in metastatic niches **(A)** Quantitative comparison of inferred interaction numbers and signaling strength between primary tumors and lymph node metastases. **(B)** Cellular interaction network within metastatic lesions, highlighting prominent connections between epithelial cells and stromal/immune components. **(C)** Circle plot depicting epithelial-centered communication with endothelial cells, fibroblasts and macrophages in metastases. **(D)** Differentially activated ligand-receptor pathways between primary and metastatic sites, including upregulated SELE and SPP1 signaling in metastases. **(E)** Network visualization of SELE-mediated cellular crosstalk in the metastatic microenvironment. **(F)** Key SELE-binding partners (CD44, GLG1) contributing to endothelial-epithelial communication. **(G)** Enhanced SELE signaling activity in TRIM26-low compared to TRIM26-high epithelial cells. **(H)** SPP1-centered interaction network among major cell populations in metastases. **(I)** Primary SPP1-mediated ligand-receptor interactions (CD44, ITGAV/ITGB1) facilitating stromal-epithelial communication. **(J)** Significantly strengthened SPP1 signaling in TRIM26-deficient epithelial populations.

CellChat analysis revealed a more complex and intense interactome in lymph node metastases compared to primary tumors, with 2,817 versus 1,675 inferred interactions and elevated overall signaling strength (Figure 8A). Within the metastatic niche, epithelial cells showed prominent communication with endothelial cells, fibroblasts, and monocytes/macrophages (Figures 8B,C). Key signaling pathways—including SELE, SPP1, ANXA1, and OSM—were preferentially activated in metastases, while CD40, LCK, and CD23 signaling dominated in primary sites (Figure 8D). Further dissection of the SELE network identified endothelial cells as the primary signal source. TRIM26-low epithelial cells exhibited significantly stronger interactions within this network than their TRIM26-high counterparts, predominantly mediated by SELE-CD44 and SELE-GLG1 ligand-receptor pairs (Figures 8E–G). Similarly, within the SPP1 axis—where macrophages acted as the main senders—communication via SPP1-CD44 and SPP1-ITGAV/ITGB1 was markedly elevated in TRIM26-low epithelial populations (Figures 8H–J). These results indicate that loss of TRIM26 expression amplifies specific stromal–epithelial and immune–epithelial communication circuits, likely promoting metastatic adaptation.

### 3.7 TRIM26 loss reshapes transcriptional networks and activates a TP53-centered regulatory hub

**FIGURE 9:**
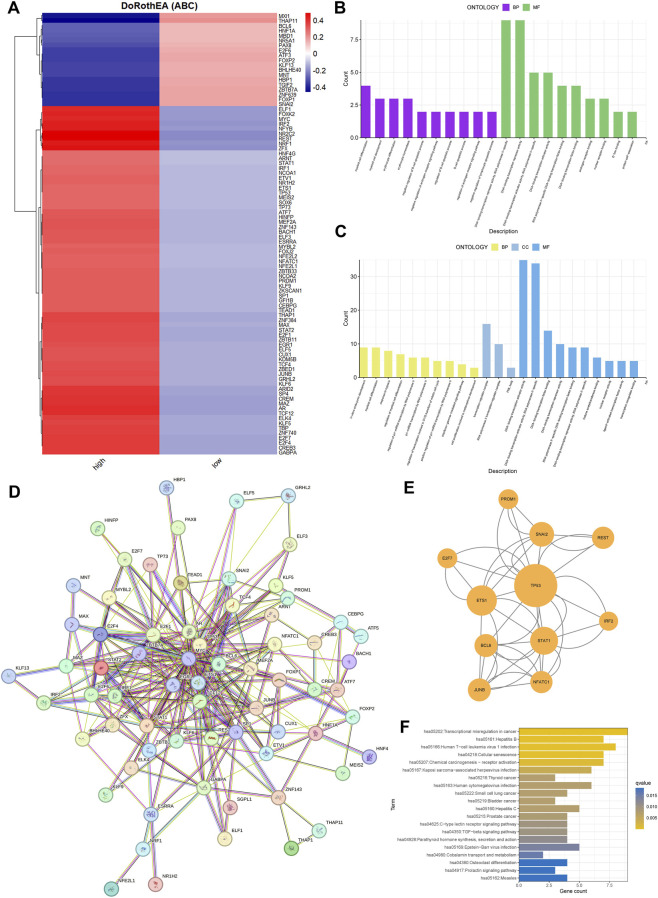
TRIM26 loss reshapes transcriptional networks and activates a TP53-centered regulatory hub. **(A)** Activity profiles of transcription factors significantly altered between TRIM26-high and TRIM26-low epithelial cells. **(B)** Gene Ontology terms enriched among transcription factors with elevated activity in TRIM26-deficient cells. **(C)** Biological processes associated with transcription factors showing reduced activity in TRIM26-low populations. **(D)** Protein-protein interaction network comprising transcription factors with TRIM26-associated activity changes. **(E)** Core regulatory subnetwork organized around TP53, identified from the comprehensive PPI analysis. **(F)** KEGG pathway analysis of differentially active transcription factors.

Comparative transcription factor (TF) activity profiling identified 90 TFs with differential regulation between TRIM26-high and TRIM26-low epithelial cells. TRIM26-deficient populations showed elevated activity of MXI1, THAP11, BCL, and HNF1A, alongside suppressed activity of ELF1, FOXP1, MYC, IRF2, and NFYB (Figure 9A). Functional annotation of upregulated TFs highlighted enrichments in differentiation programs, stress adaptation, immune modulation, and chromatin remodeling (Figure 9B), while downregulated TFs were linked to hematopoietic development, lymphocyte apoptosis, and hormonal signaling (Figure 9C). Protein-protein interaction reconstruction uncovered a tightly connected subnetwork orchestrated by TP53, comprising 11 core regulators such as ETS1, STAT1, BCL6, and SNAI2 (Figures 9D,E). This TP53-driven module likely constitutes a transcriptional mechanism through which TRIM26 depletion promotes GC progression and metastatic dissemination. KEGG pathway analysis further connected these TF changes to immune regulation, cell fate determination, and cellular senescence (Figure 9F).

### 3.8 TRIM26 deficiency drives a pro-metastatic metabolic phenotype

**FIGURE 10:**
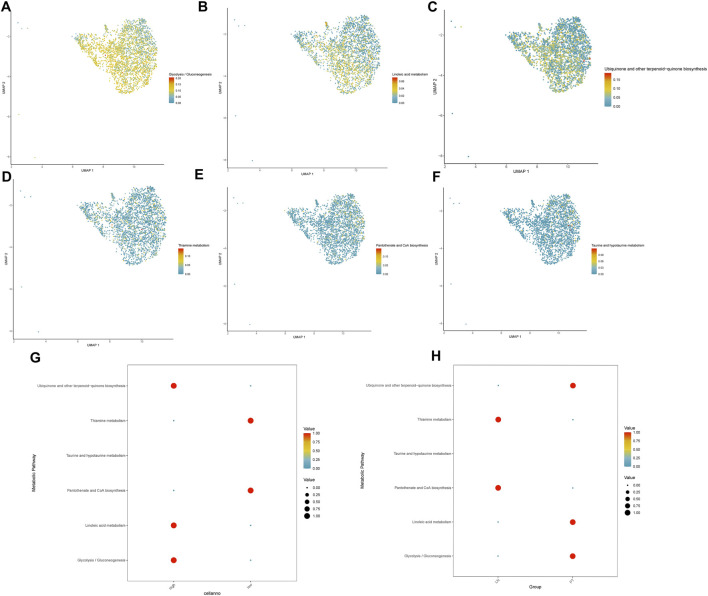
TRIM26 deficiency drives a pro-metastatic metabolic phenotype **(A)** Enhanced taurine and hypotaurine metabolic flux in TRIM26-low GC cells. **(B)** Upregulated pantothenate and CoA biosynthetic pathway supporting cofactor supply and lipid metabolism. **(C)** Activated thiamine metabolism facilitating energy production and nucleotide synthesis. **(D)** Suppressed glycolytic/gluconeogenic flux in TRIM26-deficient populations. **(E)** Impaired linoleic acid metabolism indicating altered lipid processing capacity. **(F)** Reduced ubiquinone and terpenoid-quinone biosynthesis affecting electron transport chain function.

Metabolic reprogramming analysis identified significant alterations in 16 core pathways associated with TRIM26 expression levels. TRIM26-low cells exhibited marked upregulation of nine metabolic processes, most prominently taurine/hypotaurine metabolism (Figure 10A), pantothenate and CoA biosynthesis (Figure 10B), and thiamine metabolism (Figure 10C). Conversely, seven pathways were substantially suppressed, including glycolysis/gluconeogenesis (Figure 10D), linoleic acid metabolism (Figure 10E), and ubiquinone biosynthesis (Figure 10F).

The coordinated enhancement of taurine, pantothenate/CoA, and thiamine metabolic fluxes—the three most significantly altered pathways—suggests a metabolic state optimized for antioxidant defense, cofactor supply, and energy maintenance (Figures 10G,H). This reprogramming pattern implies that TRIM26 loss activates a metabolic adaptation strategy that supports tumor cell survival and dissemination within the metastatic microenvironment.

### 3.9 Structural and functional evidence supports TRIM26 as a predictive biomarker for gemcitabine and paclitaxel sensitivity

**FIGURE 11:**
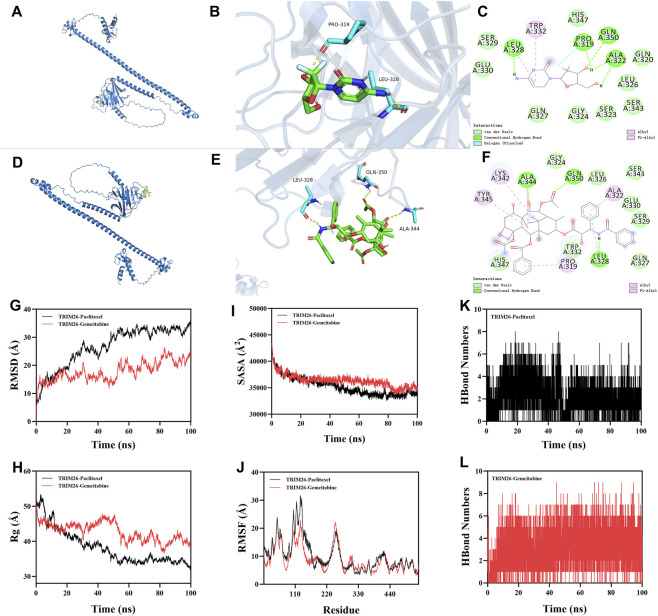
Structural and functional evidence supports TRIM26 as a predictive biomarker for gemcitabine and paclitaxel sensitivity. Three-dimensional structure of gemcitabine (purple) docked into the TRIM26 binding pocket. **(B)** Surface representation of TRIM26 with gemcitabine embedded within the complementary binding cavity. **(C)** Two-dimensional interaction map illustrating molecular contacts between gemcitabine and TRIM26 residues, including hydrogen bonds and a distinctive halogen bond. **(D)** Predicted binding conformation of paclitaxel (orange) within the TRIM26 active site. **(E)** Molecular surface depiction of TRIM26 accommodating paclitaxel within its binding cleft. **(F)** Interaction diagram detailing hydrogen bonding and hydrophobic contacts mediating paclitaxel-TRIM26 recognition. **(G)** Backbone root mean square deviation (RMSD) trajectories for TRIM26-drug complexes throughout 100 ns molecular dynamics simulations. **(H)** Time-dependent variation in intermolecular hydrogen bond numbers between TRIM26 and each ligand. **(I)** Radius of gyration (Rg) profiles indicating conformational compaction during simulation. **(J)** Solvent accessible surface area (SASA) changes reflecting dynamic surface remodeling. **(K,L)** Residue-specific root mean square fluctuation (RMSF) patterns for **(K)** TRIM26-paclitaxel and **(L)** TRIM26-gemcitabine complexes, highlighting key binding regions.

Computational chemosensitivity analysis using the pRRophetic algorithm on TCGA data demonstrated that low TRIM26 expression correlates significantly with increased predicted IC_50_ values for gemcitabine (P < 0.001) (and paclitaxel (P = 0.015)), indicating reduced drug sensitivity *in silico*. No comparable association was observed for cisplatin or docetaxel (P > 0.05), supporting the potential utility of TRIM26 as a selective predictive biomarker for these two agents (Supplementary Figure S1). To investigate a potential structural basis for this specificity, we performed molecular docking and dynamics simulations. Docking revealed a predicted stable binding mode for both drugs within a defined TRIM26 pocket, with calculated binding free energies of −6.7 kcal/mol (paclitaxel) and −5.5 kcal/mol (gemcitabine). Paclitaxel was predicted to form hydrogen bonds with ALA344, GLN350, and LEU328, complemented by extensive hydrophobic interactions. Gemcitabine was predicted to establish hydrogen bonds and hydrophobic contacts with PRO319 and LEU328, along with a distinctive halogen bond through its fluorine atom to PRO319 (Figures 11A–F). During 100 ns molecular dynamics simulations, both complexes maintained structural stability. This was evidenced by low and convergent root mean square deviation (RMSD), persistent intermolecular hydrogen bonding (averaging five and seven bonds for paclitaxel and gemcitabine, respectively), and stable profiles for the radius of gyration (Rg) and solvent accessible surface area (SASA), which together suggest ligand-induced conformational adaptation (Figures 11G–L). These computational findings provide a mechanistic hypothesis, suggesting that direct target engagement could be a potential basis for the drug-specific sensitivity associated with low TRIM26 expression.

### 3.10 TRIM26 overexpression reduces gemcitabine resistance and inhibits malignant via TGF-β signaling phenotypes in GC

**FIGURE 12:**
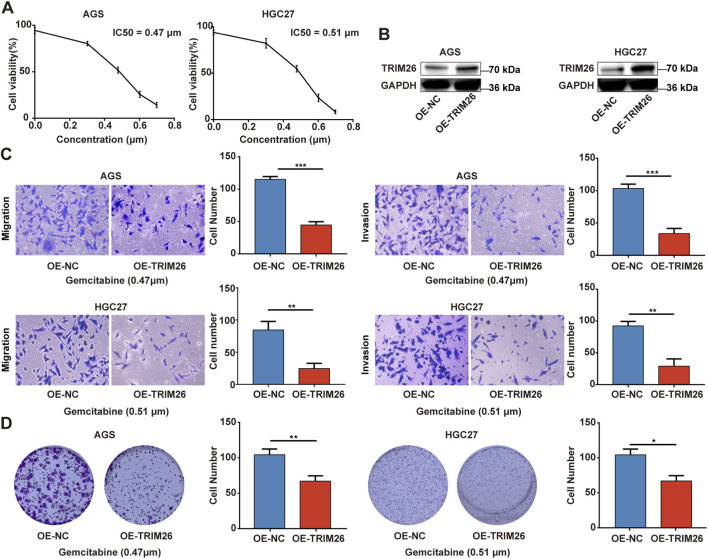
TRIM26 overexpression reverses gemcitabine resistance and inhibits malignant phenotypes in GC **(A)** Dose-response curve and IC_50_ determination for gemcitabine in GC cell lines using CCK-8 assay. **(B)** The protein expression of TRIM26 in TRIM26-overexpression AGS and HGC27 cells after infection with lentivirus carrying TRIM26. GAPDH served as a loading control. **(C)** Transwell assays were used to measure the migration and invasion of TRIM26-overexpressing AGS and HGC27 cells. **(D)** Colony formation capacity of GC cells with TRIM26 overexpression. *P < 0.05, **P < 0.01, and ***P < 0.001.

**FIGURE 13:**
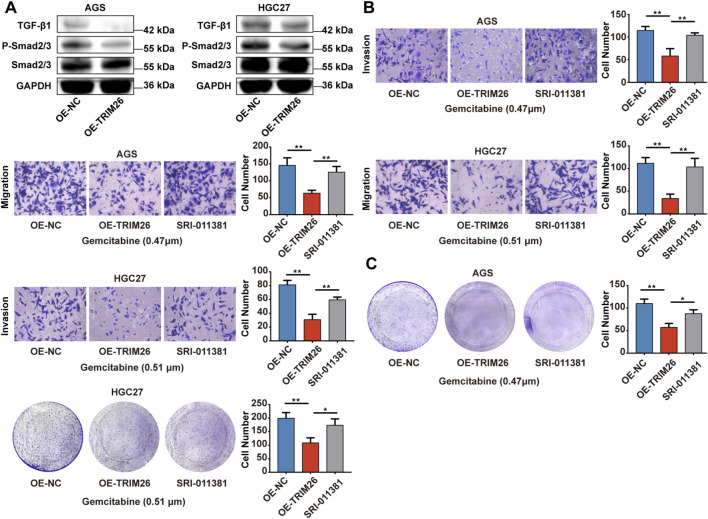
TRIM26 overexpression reverses gemcitabine resistance and inhibits malignant via TGF- βsignaling phenotypes in GC **(A)** The protein expression of TGF-β, P-Smad2/3, Smad2/3 in TRIM26-overexpression AGS and HGC27 cells. GAPDH served as a loading control. **(B)** Transwell assays were used to measure the migration and invasion of TRIM26-overexpressing AGS and HGC27 cells treated with SRI-011381 (10 μm). **(C)** Colony formation capacity of TRIM26-overexpressing AGS and HGC27 cells treated with SRI-011381 (10 μm). *P < 0.05, **P < 0.01, and ***P < 0.001.

In order to explore the potential biological functions of TRIM26 and its association with gemcitabine resistance in a more thorough manner, we conducted an experimental verification of the gemcitabine IC_50_ values of AGS and HGC27 using the CCK-8 (Figure 12A). Next, we constructed TRIM26-overexpressing stable GC cell lines. (Figure 12B). As shown in Figure 12C, TRIM26-overexpressed cells exhibited markedly reduced invasive and migratory potential in Transwell assays. Concurrently, TRIM26 overexpression significantly enhanced gemcitabine-mediated growth inhibition, resulting in reduced proliferation and diminished colony-forming capacity under gemcitabine treatment (Figure 12D). To determine if the TGF-β signaling pathway is linked to the TRIM26-mediated gemcitabine resistance and malignant potential we documented earlier, we performed Western blot assays, which revealed that the expression levels of key molecules in the TGF-β signaling cascade: TGF-β1 and P-smad2/3 in TRIM26-overexpressing GC cells were markedly reduced, demonstrated that TRIM26 exerts an inhibitory effect on the TGF-β signaling pathway (Figure 13A). Subsequently, we performed a rescue assay using SRI-011381, a specific agonist of the TGF-β signaling pathway, to verify whether TRIM26 exerts its effects on gemcitabine resistance and malignant potential through modulation of the TGF-β signaling cascade. As illustrated in Figures 13B,C, the suppressed cell migration, invasion and colony formation capacities induced by TRIM26 overexpression were significantly restored following treatment with SRI-011381 (10 μm). Taken together, these findings identify TRIM26 as a functional tumor suppressor in GC, whose downregulation not only promotes aggressive cellular phenotypes but also impairs gemcitabine sensitivity, more importantly, we further confirmed that this biological function is in fact mediated through the TGF-β signaling, thereby underscoring its dual role in governing both malignant progression and chemotherapeutic responsiveness.

## 4 Conclusion

This study establishes TRIM26 as a key regulator of LNM in GC. Downregulation of TRIM26 promotes tumor progression by remodeling intercellular communication in the tumor microenvironment, activating a TP53-centered transcriptional network, and inducing metabolic reprogramming. Functional experiments demonstrated that TRIM26 overexpression significantly suppressed the proliferation, colony formation, migration, and invasion of GC cells, while clinical specimen analysis confirmed its reduced expression in tumor tissues. Pharmacological investigations revealed that TRIM26-low GC cells exhibit enhanced sensitivity to gemcitabine and paclitaxel, and computational simulations provided structural evidence that both chemotherapeutic agents stably bind to the TRIM26 protein via favorable molecular interactions. In summary, TRIM26 acts as a tumor suppressor by modulating multi-level signaling, transcriptional regulation, and metabolic processes, while its expression level may serve as a potential biomarker for predicting the efficacy of gemcitabine or paclitaxel-based chemotherapy. This dual role underscores the significance of TRIM26 in prognostic assessment and precision therapy for GC metastasis.

## 5 Discussion

As an E3 ubiquitin ligase within the TRIM family, TRIM26 engages in multifaceted regulatory mechanisms governing cell proliferation, apoptosis, DNA damage repair, and immune responses. TRIM proteins are increasingly recognized as pivotal epigenetic modulators and ubiquitin ligases that maintain genomic integrity and shape innate immune signaling (Zou et al., 2024). Numerous TRIM members display dysregulated expression in various cancers, where they influence tumorigenesis and progression through pathway modulation (Sun et al., 2023). In GC, for instance, TRIM28 facilitates proliferation by suppressing p53 activity, TRIM44 drives invasion via AKT/mTOR signaling, and TRIM59 correlates with poor prognosis, supporting its oncogenic role (Zou et al., 2024)^.^ In contrast, the functional relevance of TRIM26 in GC remains inadequately characterized. Previous reports indicate tumor-suppressive roles in cervical and hepatocellular carcinomas, where TRIM26 modulates interferon signaling, antigen presentation, and immune infiltration (Wang et al., 2023b), yet its contribution to GC LNM is unknown.

We initially hypothesized that TRIM26 might suppress metastasis through DNA damage response or immune-regulatory routes. However, integrated bioinformatics and experimental data revealed that TRIM26 is notably downregulated in lymph node metastases relative to primary gastric tumors, indicating selective repression during metastatic progression. Clinically, reduced TRIM26 expression correlated with poorer overall survival and increased lymph node involvement, reinforcing its relevance in GC progression.

The TGF-β pathway exhibits a well-established duality in cancer: it constrains early tumor growth but promotes malignancy in advanced stages by inducing epithelial-mesenchymal transition (EMT), angiogenesis, and immune evasion (Tang et al., 2024; Zhang et al., 2024; Gao et al., 2022). This functional shift often results from mutational accumulation, altered expression of pathway components, and microenvironmental crosstalk (Li et al., 2023b; Katz et al., 2016). Our multi-omics analyses identified a strong inverse relationship between TRIM26 expression and TGF-β activation. In TRIM26-low tumors, SMAD3, SNAI1, and ZEB1 were markedly upregulated, suggesting that TRIM26 may negatively regulate TGF-β signaling, potentially through ubiquitin-mediated degradation of SMAD2/3. Gene set enrichment analysis further revealed EMT signatures in TRIM26-deficient cells, characterized by loss of adhesion molecules and enhanced cytoskeletal remodeling—consistent with known roles of TRIM proteins such as TRIM33/TIF1γ in modulating TGF-β/SMAD signaling (Chen et al., 2017; Wang et al., 2020).

Single-cell communication analysis demonstrated that TRIM26 loss amplifies pro-metastatic crosstalk within the tumor microenvironment. Specifically, TRIM26-low epithelial cells exhibited intensified interactions with endothelial cells and macrophages. Key activated pathways in lymph node metastases included the SELE-CD44/SLeX axis, which promotes endothelial adhesion and extravasation (Zheng et al., 2024); SPP1 signaling through CD44 and integrins, enhancing migration and polarizing macrophages toward an immunosuppressive phenotype (Sathe et al., 2023); ANXA1-FPR–mediated angiogenesis and T-cell suppression (Hou et al., 2024); and OSM–driven activation of JAK/STAT and MAPK cascades, facilitating EMT and stromal remodeling (Lee et al., 2021). Thus, TRIM26 downregulation appears to unleash a coordinated signaling network that supports vascular invasion and pre-metastatic niche formation.

Metabolic adaptation is critical for metastatic success under environmental stress (Halma et al., 2023; Kroemer and Pouyssegur, 2008). We observed that TRIM26 deficiency reprograms several metabolic pathways: taurine metabolism—implicated in osmoprotection and oxidative defense (Tufail et al., 2024; Cao et al., 2024); pantothenate/CoA biosynthesis, supporting lipid metabolism and membrane assembly (Hutschenreuther et al., 2013); and thiamine metabolism, fueling energy and nucleotide production (Peterson et al., 2020). Conversely, glycolytic and oxidative phosphorylation pathways were suppressed, indicating a shift toward metabolic plasticity that favors survival under nutrient and oxygen deprivation (Pa et al., 2023; Irshad et al., 2023). TGF- signaling has been shown to promote such metabolic rewiring via Smad and non-Smad routes (e.g., PI3K/AKT), modulating MYC and SREBP to enhance lipogenesis and stress adaptation (Peng et al., 2022; Derynck and Zhang, 2003). TRIM26 loss may thus potentiate TGF-β–mediated metabolic remodeling to bolster metastatic fitness.

Transcriptional network analysis further uncovered a TP53-centered regulatory module in TRIM26-low cells, involving ETS1, STAT1, BCL6, and SNAI2—key regulators of cell fate, EMT, and immune activity (Voskarides and Giannopoulou, 2023; Chatterjee et al., 2024; Palakur et al., 2023)^.^ As several TRIM proteins (e.g., TRIM24, TRIM28) regulate TP53 stability via ubiquitination (Ahsan et al., 2024; Vu et al., 2023), we propose that TRIM26 may similarly influence TP53 function. In TRIM26-low cells, TP53-mediated programs related to apoptosis and DNA repair were attenuated, whereas EMT and immune-evasion signatures were enriched. Cross-talk between this TP53 network and TGF-β signaling was evident: STAT1 and ETS1 form transcriptional complexes with SMADs, and SNAI2—a TGF-β–responsive EMT inducer—is indirectly regulated by TP53 (Ji et al., 2020). These interactions suggest that TRIM26 loss disrupts TP53-centered transcriptional homeostasis, thereby amplifying TGF-β–driven malignant phenotypes.

In summary, this work establishes TRIM26 as a key suppressor of lymphatic metastasis in GC and highlights its potential therapeutic relevance. Drug sensitivity analysis revealed that TRIM26-low GC cells exhibit heightened responsiveness to gemcitabine and paclitaxel in preclinical models. Computational structural analyses indicated that both drugs could stably bind to a TRIM26 pocket, with favorable binding energies of −5.5 kcal/mol (gemcitabine) and −6.7 kcal/mol (paclitaxel). Specific interactions included hydrogen bonds, hydrophobic contacts, and a distinctive halogen bond between gemcitabine and PRO319. Subsequent molecular dynamics simulations (100 ns) further supported complex stability, persistent interfacial hydrogen bonding, and ligand-induced conformational changes. These structural insights propose a model in which gemcitabine and paclitaxel may act as putative direct TRIM26 ligands, although experimental validation of this binding interaction is warranted. We hypothesize that in TRIM26-low tumors, these drugs might potentially stabilize residual TRIM26 or mimic its tumor-suppressive function, thereby restoring partial activity and exploiting vulnerabilities associated with TRIM26 loss—such as TGF-β hyperactivation and metabolic reprogramming. While traditionally recognized for targeting DNA synthesis or microtubule dynamics, the efficacy of these agents in TRIM26-deficient GC may also involve this proposed mechanism. Thus, our findings nominate TRIM26 as a candidate biomarker for predicting chemosensitivity to gemcitabine and paclitaxel. Importantly, future studies integrating Mendelian randomization approaches may help clarify the causal relationship between genetic determinants of TRIM26 expression and gastric cancer progression or therapeutic response, thereby strengthening causal inference beyond observational and experimental data (Ji et al., 2020; Zuo et al., 2023). Such genetically informed analyses, combined with prospective clinical validation, could further establish the clinical utility of TRIM26 in guiding precision therapy for GC patients.

## Data Availability

The raw data supporting the conclusions of this article will be made available by the authors, without undue reservation.
