# Supplementary figures and images for: TRIM26 deficiency drives gastric cancer lymph node metastasis via TGF-β signaling activation and modulates gemcitabine response
Source: Front Cell Dev Biol. 2026 Feb 5;14:1746425. doi: 10.3389/fcell.2026.1746425 (PMC12916669; doi:10.3389/fcell.2026.1746425)

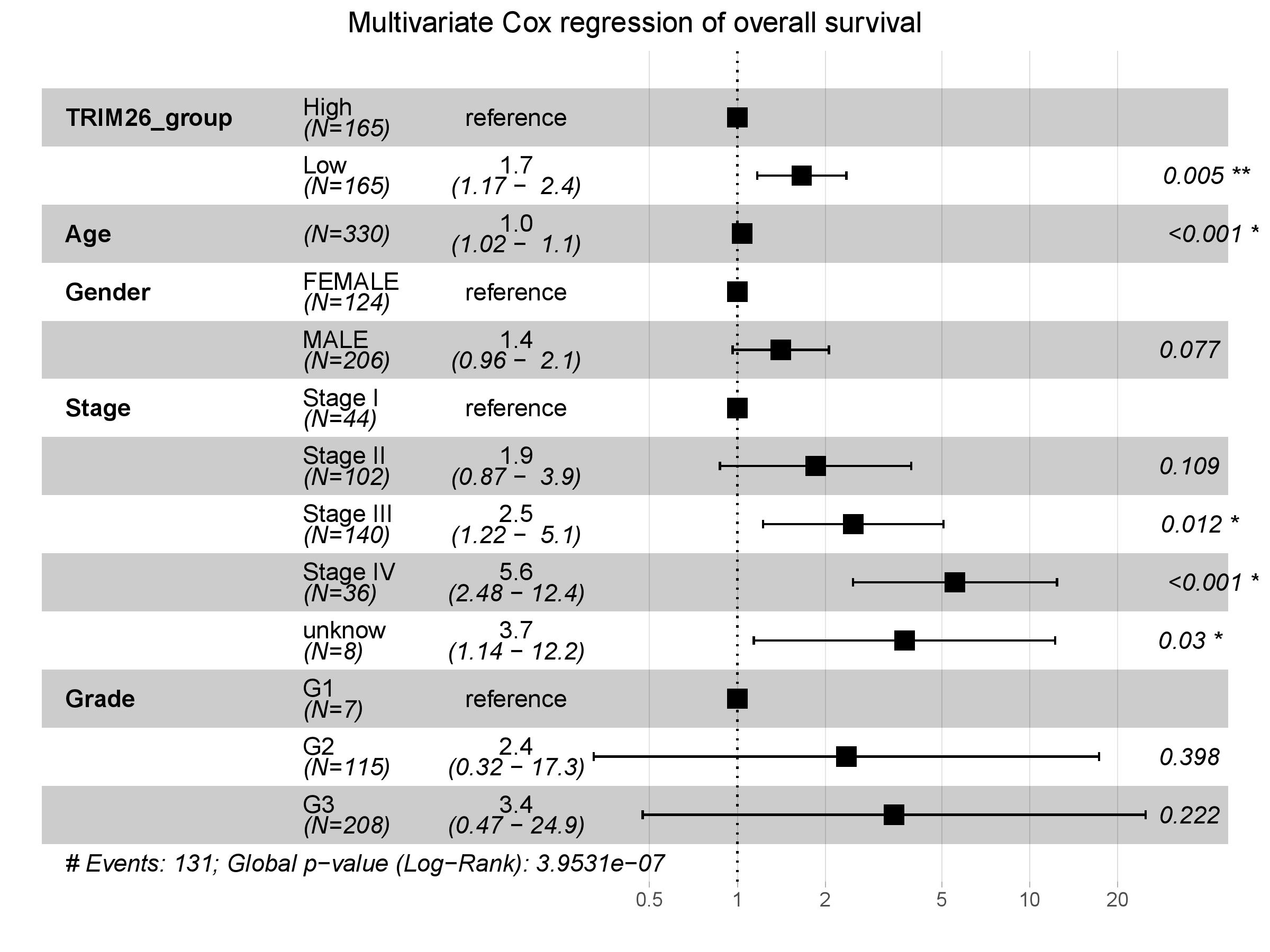

Supplement: Supplementary file 2 [file Image2.jpeg]
